# A Double-Blind, Randomized Controlled 12-Week Follow-Up Trial to Evaluate the Efficacy and Safety of Polycan in Combination with Glucosamine for the Treatment of Knee Osteoarthritis

**DOI:** 10.1155/2019/9750531

**Published:** 2019-06-24

**Authors:** Thi Thanh Thuy Truong, Jong Min Lim, Hyung-Rae Cho, Young Suk Kim, Duc Giang Dao, Quoc Hung Tran, Jae-Suk Choi

**Affiliations:** ^1^198 Hospital, No. 09 Tran Binh Street, Mai Dịch, Cau Giay, Hanoi, Vietnam; ^2^Glucan Corp., #305 Marine Bio-Industry Development Center, Hoenggye-ri 27, Ilgwang-myeon, Gijan-gun, Busan 46048, Republic of Korea; ^3^VietStar Biomedical Research, Room 2, 5th Floor, Horison Tower, 40 Cat Linh, Dong Da District, Hanoi, Vietnam; ^4^Division of Bioindustry, College of Medical and Life Sciences, Silla University, 140 Baegyang-daero, 700 Beon-gil, Sasang-gu, Busan 46958, Republic of Korea

## Abstract

**Aim:**

The aim of the present study was to examine the efficacy and safety of Polycan, a *β*-glucan produced from the black yeast* Aureobasidium pullulans* SM-2001, in combination with glucosamine in reducing knee osteoarthritis-associated symptoms.

**Methods:**

This was a double-blind, randomized controlled trial of a formulated product composed of 16.7 mg of Polycan and 250 mg of glucosamine (Group A), 16.7 mg of Polycan and 500 mg of glucosamine (Group B), or 500 mg of glucosamine (control group) per capsule, administered as three capsules once per day over a period of 12 weeks, conducted with 100 osteoarthritis patients, aged 35–80 years. The primary outcome measure was osteoarthritis symptoms assessed by the Western Ontario and McMaster Universities Osteoarthritis Index (WOMAC) questionnaire. The secondary outcome measures included rescue medication use (according to data from a patient-reported diary) and other safety indices (body weight, blood pressure, hematological, and biochemistry markers).

**Results:**

Compared with the control group, Group B demonstrated a statistically significant reduction in the total WOMAC score after 12 weeks of treatment (*p* < 0.05). There was a significant reduction in the frequency of rescue medication used in Groups A and B compared with the control group (*p* < 0.05). There were no significant changes in hematology and biochemistry parameters or health indices between the active and the control group.

**Conclusion:**

Among patients with mild or moderate osteoarthritis, a daily oral dose of Polycan (50 mg) in combination with glucosamine (750 mg or 1500 mg; Group A or B, respectively) resulted in a better treatment outcome than treatment with glucosamine (1500 mg) alone.

## 1. Introduction

Knee osteoarthritis is a degenerative and chronic disease of the knee joint and is the most common type of arthritis, affecting approximately one-third of people aged over 40 years in Western countries [1, 2]. It is characterized by local joint inflammation that can progress to severe tissue damage if untreated. Knee osteoarthritis affects patients' activities of daily living to varying degrees [3, 4] and is accompanied by increased morbidity [5].

Nonsteroidal anti-inflammatory drugs (NSAIDs) are often used for the treatment of painful symptoms and inflammation. However, NSAIDs are associated with serious cardiovascular and digestive side effects and are therefore unsuitable for long-term use. Therefore, additional treatment options, with a more favorable side effect profile than that of NSAIDs, are being actively sought out to target the pain and inflammation of osteoarthritis. Further research is also required to develop pharmacological therapies that target the structural changes in osteoarthritis.

Owing to the safety concerns and side effects of NSAIDs described above, patients have turned to dietary supplements as they are a safer long-term alternative for the management of osteoarthritis symptoms. Various nutritional supplements are available for osteoarthritis, and supplements containing glucosamine and chondroitin sulphate have been shown to provide pain relief [6]. On the other hand, a recent network meta-analysis of the effects of glucosamine and chondroitin in patients with osteoarthritis of the hip or knee demonstrated that glucosamine, chondroitin, and their combination do not reduce joint pain or have an impact on joint space narrowing compared with placebo [7].

Glucosamine sulphate is the sulphate derivative of the natural amino monosaccharide glucosamine [8]. Glucosamine, a normal constituent of glycosaminoglycans in the cartilage matrix and synovial fluid, is postulated to have various pharmacological actions in articular cartilage and joint tissues. Several clinical trials and meta-analyses have shown a significant symptom-modifying effect of glucosamine sulphate and good safety profile in patients with osteoarthritis [9–11]. In a randomized, double-blind, placebo-controlled study, Pavelká et al. [10] demonstrated that long-term (3 years) treatment with glucosamine sulphate (1500 mg once a day) halted the progression of knee osteoarthritis. In addition, a randomized placebo-controlled clinical trial demonstrated the long-term (3 years) combined structure-modifying and symptom-modifying effects of glucosamine sulphate (1500 mg once a day), suggesting its clinical utility as an osteoarthritis-modifying agent [9].

Polycan, a fermentation product of the black yeast* Aureobasidium pullulans* SM-2001, consists of extracellular polysaccharides comprising a (1,3)-*β*-D-glucan backbone with a single (1,6)-*β*-D-glucan unit, as the main functional component, and other water-soluble nondigestible polysaccharides [12]. Polycan is considered a Generally Recognized As Safe (GRAS Notice No. GRN 000309) product and is globally used in the food and health food industry.

Previous animal studies have demonstrated a beneficial effect of Polycan on anterior cruciate ligament transection and partial medial meniscectomy-induced-osteoarthritis [13] and surgery-induced osteoarthritis [14]. Kim et al. [13] demonstrated that 84 days of continuous oral treatment with Polycan led to lesser degrees of articular stiffness and histological cartilage damage compared with osteoarthritis controls 91 days after osteoarthritis inducement, suggesting that the optimal Polycan dosage to treat osteoarthritis is 42.5 mg/kg/day in anterior cruciate ligament transection and partial medial meniscectomy-induced-osteoarthritis rats. According to the results of Choi et al. [14], anti-OA effects including the induction of chondrocyte proliferation were detected in 100 mg Polycan-treated group compared with those of the osteoarthritis control group in surgery-induced osteoarthritis rats.

A few studies to date have also investigated the effects of Polycan in humans. For example, one clinical study evaluated its effect on bone metabolism in healthy perimenopausal women [15] and another on bone biomarkers in healthy women [16]. To investigate the effect of Polycan on bone metabolism, Kim et al. [15] carried out a 12-week, randomized, double-blind, placebo-controlled clinical trial in Korean women. Sixty subjects were randomly assigned to two treatment groups with daily doses of Polycan (n=30, 150 mg) or placebo (n=30, 150 mg). After the 12 weeks of supplementation, osteocalcin showed a tendency to increase in the Polycan group, therefore suggesting that Polycan has positive effects on bone metabolism. Kim et al. [16] demonstrated that administration of Polycan (400 mg once daily) for 28 days in healthy women resulted in modest inhibition of the increase in CTx. No statistically significant effect of Polycan was seen on other biomarkers of bone metabolism.

Previous studies have reported a synergistic effect of glucosamine and other compounds, such as chondroitin, on knee osteoarthritis [6, 17, 18].* In vitro* studies have demonstrated a synergistic effect of a Polycan and calcium lactate–gluconate mixture on osteoclast and osteoblast activity [19], and similar effects have been demonstrated in rats with surgery-induced osteoarthritis [14]. However, a synergistic effect of glucosamine and Polycan on knee osteoarthritis has not been confirmed.

Therefore, in this clinical study, we examined the efficacy and safety of Polycan in combination with glucosamine in reducing knee osteoarthritis-associated symptoms in adults with mild to moderate osteoarthritis. Efficacy was determined in a double-blind, randomized controlled trial conducted with 100 osteoarthritis patients, aged 35–80 years, using formulated products.

## 2. Materials and Methods

### 2.1. Materials

Glucosamine was purchased from Yangzhou Rixing Bio-Tech Co. Ltd. (type: glucosamine hydrochloride; Jiangsu, Yangzhou, China). Polycan was kindly provided by Glucan Corporation (type: Polycan PN-101; Busan, Korea). Products were encapsulated by BV Pharma (Ho Chi Minh City, Vietnam) using gelatin capsules size 0.

### 2.2. Study Design

**Figure 1:**
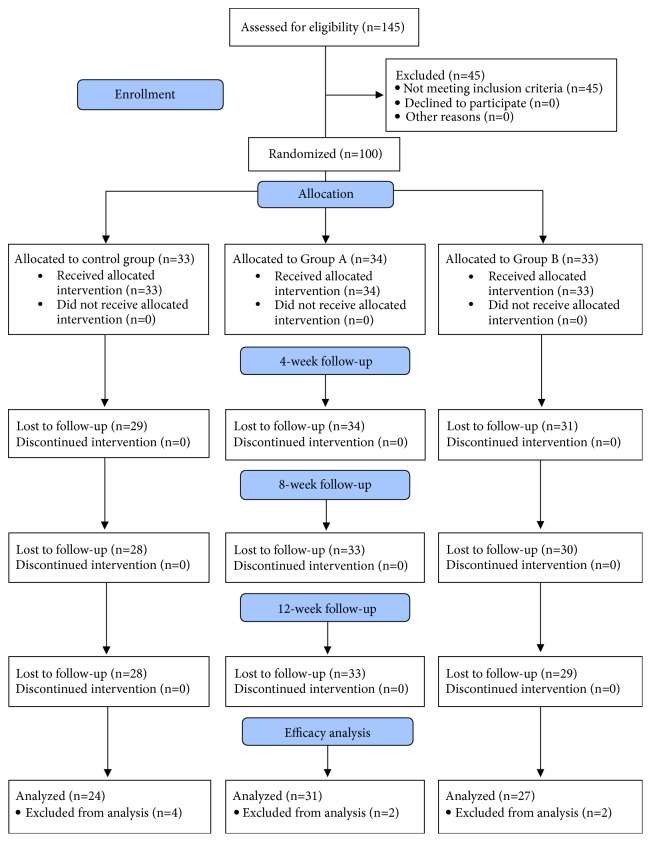
Patient enrolment and follow-up protocol.

This was a single-site, double-blind, three-parallel-group, randomized controlled clinical trial of 12 weeks' duration to assess the safety and efficacy of Polycan in combination with glucosamine in reducing symptoms of knee osteoarthritis in patients with severity of osteoarthritis ranging from grade 1 to grade 3 by the Kellgren and Lawrence system [20] (Figure 1 and Table 1). The study was conducted between April 2017 and May 2018 at A8 Internal Department, 198 Hospital, Hanoi, Vietnam. The study was conducted in accordance with the Principles of the Declaration of Helsinki and was approved by the Research Ethics Committee of 198 Hospital.

#### 2.2.1. Inclusion Criteria

Male and female patients who met the following inclusion criteria were enrolled: (1) age 18 to 80 years at the time of enrolment; (2) a diagnosis of osteoarthritis according to the Guidelines for Diagnosis of Osteoarthritis of the National Clinical Guideline Centre [21]; (3) osteoarthritis of knee joints grade I, II, or III according to the Kellgren and Lawrence criteria [20]; and (4) no contraindications for oral NSAIDs. All patients provided written informed consent prior to study enrolment.

#### 2.2.2. Exclusion Criteria

Patients were excluded if they (1) had a history of hypersensitivity to any of the substances in the investigational products or to NSAIDs; (2) were pregnant or breastfeeding; (3) were unable to undergo knee joint ultrasound or radiographic examination; (4) were eligible for surgical treatment or joint replacement, as determined by the investigator; and (5) had other conditions which, according to the investigator's opinion, made them ineligible for the study treatment.

The primary efficacy outcomes were the absolute reduction in overall WOMAC scores, including pain, stiffness, and function subscale scores, from baseline [22]. Secondary outcome measures were meloxicam use; nature, onset, duration, severity, and outcome of all adverse events at each visit; and hematology (full blood count), biochemistry indices (alanine aminotransferase, aspartate aminotransferase, creatinine, albumin, total bilirubin, glucose, urea, and total protein), BMI, and vital signs. WOMAC scores were recorded at baseline and after 4, 8, and 12 weeks of treatment [22].

Patients were provided with a diary and instructed to record self-scored knee pain as well as adverse events over the course of the study. At study visits, they were asked to report symptoms of highest severity that they suffered in the preceding four weeks.

In this double-blinded study, all staff members and patients involved were unaware of the group assignments. Patients were provided with study drug packages matching with their randomization number.

### 2.3. Study Treatments and Oversight

Patients were randomly assigned in a 1:1:1 ratio to take 500 mg of glucosamine (control group), 16.7 mg of Polycan and 250 mg of glucosamine (Group A), or 16.7 mg of Polycan and 500 mg of glucosamine (Group B) per capsule, administered as three capsules once per day over a period of 12 weeks. The proper amount of dextrin, as an excipient, was added to the capsule of Group A and to the control group to equalize the weight of the capsules in each group. All pain medications except the “rescue” medication were discontinued at the screening visit.

The study medication was administered orally on a daily basis. Patients were instructed to take 3 capsules of the investigational product, once a day every day, with water. There were no restrictions on food consumption in this study. The study protocol did not require patients to take the IMP (impression) at a particular time of the day. Study visits were scheduled for week 4, week 8, and week 12, during which safety and efficacy assessments were performed and serum samples for routine laboratory tests were obtained. In addition, patients were contacted by telephone every two weeks to ask about adverse events. Patients recorded their knee pain and their use of rescue medication daily in an outpatient diary. The only rescue medication that was permitted was meloxicam at a dose of 7.5 mg/day. Patients were provided with a prescription for meloxicam by the study physician and advised to self-administer meloxicam orally as required to control their pain.

### 2.4. Statistical Analysis

Statistical analyses were conducted using SPSS for Windows (Release 14K, SPSS Inc., Chicago, IL). Primary and secondary efficacy outcomes were assessed in the per-protocol population, which included all patients who had undergone randomization and completed 12 weeks of treatment without major protocol deviation. This is a pilot study, and the sample size was indicative and was not calculated to test any study hypothesis. Nevertheless, we determined that, with 30 evaluable patients in each group, the study would have more than 80% power to detect a difference of 5 points in the WOMAC score after 12 weeks of treatment compared to the baseline, with paired* t*-tests of the log-transformed WOMAC score in each group assuming that the common standard deviation of the WOMAC at baseline and after 12 weeks of treatment is 10 points.

The Kolmogorov–Smirnov test was used to test if the continuous variables (e.g., WOMAC score, absolute reduction of WOMAC score after 12 weeks of treatment) were normally distributed or not, and the* t*-test and Wilcoxon–Mann–Whitney test were used for normally distributed and nonnormally distributed variables, respectively. For comparison between more than 3 groups, parametric ANOVA or nonparametric ANOVA (Kruskal-Wallis test) was used, respectively, for continuous variables. For categorical variables, the chi-square or Fisher exact test was used. For all the statistical test, a* p* value which is less than 0.05 would be considered as statistically significant [23].

## 3. Results

### 3.1. Trial Population

Patients were recruited from the Outpatient Department of 198 Hospital over a 6-month period from July 2017 to December 2017. One hundred and forty-five patients were examined by orthopedists for knee osteoarthritis problems and were assessed for eligibility to enter the study according to the inclusion and exclusion criteria. One hundred patients met the study criteria and were enrolled, of which 33, 34, and 33 patients were randomized into the control group (glucosamine 1500 mg per day), Group A (Polycan 50 mg and glucosamine 750 mg per day), and Group B (Polycan 50 mg and glucosamine 1500 mg per day), respectively (Figure 1). Approximately 75% of the patients in all three groups were classified as OA grade 2 and roughly 20% of the patients in all three groups were OA grade 3 by the Kellgren and Lawrence system. None of the patients in any of the study groups reported history of partial or total meniscectomy.

A total of 10 patients were lost to follow-up by the end of treatment (5, 1, and 4 patients in the control group, Group A, and Group B, respectively). Of the evaluable patients at end of treatment, 4 from the control group, 2 from Group A, and 2 from Group B were excluded from efficacy analysis because of major protocol deviation (use of prohibited concomitant medications). Thus, 24 patients who received glucosamine 1500 mg per day, 31 patients who received Polycan (50 mg per day) and glucosamine (750 mg per day), and 27 patients who received Polycan (50 mg per day) and glucosamine (1500 mg per day) were included in the per-protocol population.

Baseline demographic and clinical characteristics, including age, body mass index (BMI), sex, and WOMAC total score, were not significantly different between the groups (Tables 2(a) and 2(b)). The mean WOMAC total score at the time of the baseline examination was approximately 45 points in the three groups.

### 3.2. Efficacy Assessment

The primary endpoint in all 3 groups was change in the WOMAC score at 12 weeks. Compared to baseline, there was a statistically significant reduction in the WOMAC score in all three groups. The WOMAC total score was reduced by 13.1, 16.3, and 20.1 points in the control group, Group A, and Group B after 12 weeks of treatment, respectively (*p*<0.001). In a paired-wise comparison, there was a greater reduction in the total WOMAC score in Group B compared to the control group, and this difference was statistically significant (*p*=0.04).

**Figure 2:**
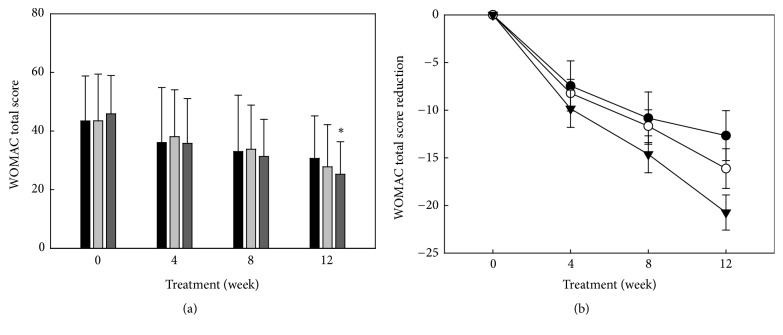
Mean value (a) and absolute change value (b) of WOMAC pain changes from baseline to 4, 8, and 12 weeks in the control group (■: (a), ●: (b); glucosamine 1500 mg per day), Group A (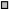: (a), ○: (b); Polycan 50 mg and glucosamine 750 mg per day), and Group B (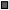: (a), ▼: (b); Polycan 50 mg and glucosamine 1500 mg per day), respectively, in this clinical study (*∗p*<0.05 compared to control).

Figure 2 shows the WOMAC total score at baseline and after 4 weeks, 8 weeks, and 12 weeks of treatment in the per-protocol population. WOMAC total scores at baseline were 43.46, 43.47, and 45.87 in the control group, Group A, and Group B, respectively. After 12 weeks of treatment, these were reduced to 30.67, 27.81, and 25.26 (*p*<0.001) in the control group, Group A, and Group B, respectively (Table 3).

Meloxicam (7.5 mg) was used as rescue medication for all three groups. Patients were asked to record every use of meloxicam. The average monthly number of dosages of rescue medication used is shown in Table 4. Patients in Group A and Group B used an average of 1.4 to 1.6 doses of meloxicam (7.5 mg) per month as rescue medication, which was statistically significantly less than that in the control group, which used approximately twice as much as Group A and Group B (Table 4).

### 3.3. Safety Assessment

In all tested groups, at week 12, there were no significant abnormal changes in hematology and clinical chemistry compared to baseline. There were no remarkable changes in the complete blood counts, differential white blood cell counts, hepatic and renal functions, lipid profiles, BMI, and vitals in all three groups. Three adverse events were reported in Group A: facial edema (n=1), headache (n=1), and hypertension (n=1). In all cases, the adverse events were confirmed as unrelated to the investigational products.

## 4. Discussion

Osteoarthritis is a major public health problem for which there are few effective medical remedies [24]. Nonsteroidal anti-inflammatory agents are the most commonly prescribed agents for this disorder but can be associated with serious side effects [25].

Glucosamine is highly effective in osteoarthritis, and Polycan is effective in osteoarthritic animal models [9, 10, 13, 14]. However, no study to date has investigated whether these two compounds have a synergistic benefit for patients with osteoarthritis. Therefore, in this study, the synergistic effects of these two materials were evaluated in a double-blind, randomized controlled, 12-week follow-up intervention clinical study.

In this trial involving mild to moderate osteoarthritis patients, the functional outcome at week 12 measured by WOMAC was more favorable among the patients who received Polycan (50 mg) plus glucosamine (750 mg or 1500 mg; Group A or B, respectively) than among those who received glucosamine (1500 mg). WOMAC reduction was chosen as the primary efficacy endpoint in this study as it is widely accepted in osteoarthritis studies. In this clinical study, the control group, Group A, and Group B showed changes in the WOMAC total score at week 12 compared to the baseline, and the observed changes were 13.1, 16.3, and 20.1, respectively. Group B showed a statistically significantly larger improvement than the control group in WOMAC total score reduction (Group B vs. control group:* p*= 0.04), in a paired-wise comparison (Table 3). The study was concluded after 12 weeks, but it can be appreciated that further improvements in WOMAC scores might be obtained with continued intake of Polycan in combination with glucosamine (Figure 2). Further studies with longer duration would be necessary to confirm this assumption. In addition, a daily dose of 50 mg of Polycan in combination with glucosamine was found to be associated with statistically lower usage of rescue medication than supplementation with glucosamine alone.

There seems to be no synergistic effect between glucosamine and chondroitin. Sawitzke et al. [26] evaluated the efficacy and safety of glucosamine and chondroitin sulphate, alone or in combination, as well as celecoxib and placebo on painful knee osteoarthritis. For over 2 years, glucosamine and celecoxib showed beneficial but not significant trends, in WOMAC-rated pain and function, as compared with placebo. In addition, no apparent synergy has been observed in the glucosamine and chondroitin sulphate combination-treated group.

The synergistic effects of Polycan and other materials containing calcium on osteoporosis have been previously demonstrated. Polycan and calcium lactate–gluconate (1:9, w/w) were shown to have synergistic antiosteoporotic effects* in vitro* [19]. In addition, a dose of 400 mg Polycan and calcium lactate–gluconate (1:9, w/w) has been proven to improve bone metabolism and was well tolerated and safe in a 4-week open-label clinical study [27]. However, no previous studies have evaluated the synergistic relationship between mixtures of Polycan and other materials in osteoarthritis.

In this study, it was not possible to verify whether synergistic effects of glucosamine and Polycan complex on the treatment of knee osteoarthritis exist. However, the therapeutic effect of a relatively small amount of glucosamine (750 mg) and Polycan (50 mg) mixture on knee osteoarthritis was found to be almost equivalent to that of a large amount of glucosamine (1500 mg) alone. This means that 750 mg of glucosamine can probably be replaced by 50 mg of Polycan. The treatment effect of Polycan among patients with mild or moderate osteoarthritis of the knee included in our trial is consistent with its pharmacological effects which have been demonstrated in animal models [13, 14].

There were no serious adverse events which were related to the investigational products in this study. This finding was similar to that reported in a systematic quality assessment and meta-analysis which determined that glucosamine is a safe compound [11], while Polycan was considered as “Generally Recognized As Safe (GRAS)” in a recent safety review [15, 16].

In conclusion, among patients with mild or moderate osteoarthritis, daily Polycan (50 mg) in combination with glucosamine (750 mg or 1500 mg; Group A or B, respectively) taken orally resulted in a better treatment effect than single treatment with 1500 mg of glucosamine (control group). In addition, our trial provides evidence of a clinical benefit of treatment with a glucosamine and Polycan complex in patients with mild to moderate osteoarthritis who would not usually be eligible for knee replacement.

## Figures and Tables

**Table 1:** Grading scales for the radiographic osteoarthritis classification system [20].

Grade	Characteristics
0	No joint space narrowing (JSN) or reactive changes
1	Doubtful JSN, possible osteophytic lipping
2	Definite osteophytes, possible JSN
3	Moderate osteophytes, definite JSN, some sclerosis, possible bone-end deformity
4	Large osteophytes, marked JSN, severe sclerosis, definite bone-end deformity

**Table:** (a) Patient baseline characteristics (intention-to-treat population)

		Control	Group A	Group B	*p* value
Age (years)	n	33	33	34	*p*=0.34*∗*
	Mean±SD	61.4±7.1	58.2±8.9	60.1±9.5	
	Min-Max	48-80	35-78	38-76	

BMI	n	33	33	34	*p*=0.84*∗*
	Mean±SD	22.9±2.7	23.1±2.0	23.1±2.9	
	Median	22.6	23.1	22.6	

WOMAC total score	n	33	33	34	*p*=0.99*∗*
	Mean±SD	44.9±15.2	44.4±16.2	45.2±13.1	
	Median	48	47	42	

Sex					

Male	n	2	3	3	*p*=0.9*∗∗*
	%	6.1	9.1	8.8	
Female	n	31	30	31	
	%	93.9	90.9	91.2	

*∗*: Kruskal-Wallis test. *∗∗*: Fisher exact test

**Table:** (b) Patient baseline characteristics (per-protocol population)

		Control	Group A	Group B	*p* value
Age (years)	n	24	31	27	*p*=0.06*∗*
	Mean±SD	62.8±7.6	60.3±8.8	59.6±8.8	
	Min-Max	48-80	40-76	35-78	

BMI	n	24	31	27	*p*=0.66*∗*
	Mean±SD	23.2±2.7	23.1±2.0	22.9±2.5	
	Median	23.0	23.1	22.6	

WOMAC total score	n	24	31	27	*p*=0.88*∗*
	Mean±SD	43.4±15.3	43.9±15.9	46.0±13.4	
	Median	47	46	45	

Sex					

Male	n	2	2	3	*p*=0.89*∗∗*
	%	8.33	6.45	11.11	
Female	n	22	29	24	
	%	91.67	93.55	88.89	

*∗*: Kruskal-Wallis test. *∗∗*: Fisher exact test

**Table 3:** WOMAC total score changes at week 12 compared to the baseline.

Group	Control group	Group A	Group B
N	24	31	27
Mean±SD	- 13.1±12.8	- 16.3±11.6	- 20.1±9.5
*p* value (compared to the baseline)	<0.001	<0.001	<0.001
*p* value (paired-wise comparison)	Group B vs. control group: *p*= 0.04		
*p* value (Kruskal-Wallis test to compare between 3 groups)	*p*=0.07		

**Table 4:** Usage of rescue medication (meloxicam 7.5 mg/day) in three groups.

Group	Control	Group A	Group B
N	24	31	27
Mean±SD	3.1±4.6	1.4±3.8	1.6±4.0
*p* value (compared to control)		0.001	0.02

## Data Availability

The data used to support the findings of this study are available from the corresponding author upon request.
